# Financial strain, digital health reliance, and medical preferences: a multi-dimensional analysis of Chinese residents

**DOI:** 10.3389/fpubh.2026.1829939

**Published:** 2026-05-20

**Authors:** Hejing Zhang, Yunchao Cai, Xuao Chen

**Affiliations:** School of Economics and Management, Ankang University, Ankang, China

**Keywords:** China, digital health reliance, financial strain, health related outcome, medical preferences

## Abstract

**Introduction:**

While the link between financial strain and poor health is well-established, research remains fragmented across isolated indicators and lacks an integrated understanding of how resource scarcity reshapes health-seeking strategies, particularly the use of digital resources and traditional medical systems in non-Western contexts.

**Methods:**

Utilizing data from the 2021 China General Social Survey (CGSS), this study examines the relationship between financial strain and three connected domains. The empirical analysis employs Ordinary Least Squares (OLS) regression models to analyze two independent sub-samples of working-age respondents (*N* = 623 and *N* = 592). Assessed variables span physical and psychological health outcomes, preventative behaviors, digital health reliance, and medical system preferences.

**Results:**

Regression analyses reveal that financial strain is a pervasive stressor that compromises multiple domains of well-being and is associated with significantly lower preventative health investments. In the digital sphere, financial strain is closely linked to a unique reliance on internet-based health resources, which is often accompanied by a lack of regard for information reliability. Moreover, financial strain is significantly associated with a stronger cognitive preference for, and greater behavioral utilization of, Traditional Chinese Medicine (TCM).

**Discussion:**

These findings highlight a unique adaptation strategy where individuals navigate resource scarcity by leveraging digital tools and traditional medical systems. These results have critical implications for health equity, underscoring the need for policies that address digital health literacy and integrate culturally trusted systems into formal primary care.

## 1 Introduction

Decades of research have established a robust social gradient in health, often linking lower socioeconomic status to higher rates of morbidity and mortality (1, 2). Within this framework, financial strain, specifically characterized by debt and economic hardship, has emerged as a distinct and potent stressor (3). Evidence across diverse populations suggests that financial strain acts as a chronic stressor that infiltrates multiple health domains, including physical health, mental well-being, and health-related behaviors (4–6). However, while these individual associations are well-documented, most of the literature remains fragmented, focusing primarily on isolated health indicators. This lack of an integrated perspective makes it difficult for researchers and practitioners to assess the cumulative effects of financial strain across multiple health domains.

In addition, while the internet has become a ubiquitous health resource (7), the role of financial strain in shaping digital health behaviors remains under-explored. Previous research has predominantly viewed the internet as a neutral tool for health information (8). However, from the perspective of Scarcity Theory, the internet may function as a strategic low-cost alternative for those facing financial constraints. When formal medical consultation becomes a financial burden, individuals may turn to digital triage and online self-diagnosis. Whether financial strain fosters a distinctive reliance on digital health information as a health-seeking strategy is a question of significant relevance in the digitalized era (9, 10).

Moreover, given the unique socio-cultural and healthcare context in China, more research is needed to account for socio-cultural variations and to assess how these relationships differ across various health metrics. In the Chinese context, financial strain may not merely create a barrier to healthcare; it may correspond to a preference pattern in how individuals choose between different medical systems. In China, Traditional Chinese Medicine (TCM) offers a culturally resonant and economically flexible alternative to Western medicine (11). Yet it remains unclear whether financial scarcity is associated with a behavioral preference for TCM as an alternative for health management. Understanding this is crucial for capturing the unique ways Chinese residents navigate health challenges under economic pressure.

Therefore, the present study aims to addresses these gaps by utilizing data from the China General Social Survey (CGSS) to achieve three primary objectives. The first objective of this study is to provide a holistic validation of the multi-dimensional relationships between financial strain and health among individuals in China. By utilizing two distinct yet unified modules from CGSS, this study simultaneously examines the associations between financial strain and a wide spectrum of health outcomes, including self-rated health, physiological functioning, psychological distress, sleep quality, and preventative health behaviors. This integrative approach allows us to provide a baseline, suggesting that financial strain may act as a stressor associated with most facets of an individual's well-being in the Chinese context.

Building upon this baseline, the second objective is to explore how financial strain relates to health-seeking strategies in the digital era and whether these strategies can serve as a buffer against health erosion. Moving beyond the traditional view of the internet as a mere information base, we conceptualize digital health engagement as a strategic response to resource scarcity. Under the framework of Scarcity Theory, we investigate whether financial strain is associated with greater reliance on internet-based medical triage. Specifically, we analyze whether those facing financial constraints develop a reliance on digital resources, perceiving them as an accessible alternative to professional consultation.

Finally, this study situates these health consequences and digital strategies within the specific cultural and institutional context of China by examining the varying utilization of TCM. By comparing the utilization of Western medicine vs. TCM, this study examines whether financial scarcity correlates with a higher likelihood of utilizing TCM as a culturally resonant and economically flexible alternative. This analysis reveals how the “tunneling effect” of scarcity corresponds to a re-alignment of medical preferences, where TCM may emerge as culturally resonant and economically practical alternative when formal Western medical resources become financially burdensome.

## 2 Literature review and hypotheses development

### 2.1 Financial strain and health depletion

To explain why various stressors have differing effects on an individual's well-being, many researchers rely on the Stress Process Model as a prominent framework (12, 13). Being a foundational sociological framework, this model posits that financial strain functions as a primary stressor rooted in social stratification. Crucially, it emphasizes stress proliferation where economic hardship creates a cascade of secondary stressors, such as the erosion of personal mastery and self-esteem, that wear down an individual's psychological defenses. However, while the Stress Process Model powerfully illustrates how structural disadvantages lead to distress, it offers a macro-sociological framework. Later Mani et al. (14) in their significant study specifically explained how scarcity affects individuals' mental health at the micro level.

According to the Scarcity Model (14), financial scarcity directly consumes an individual's mental bandwidth, leading to declines in cognitive function and executive control. This chronic psychological burden leads to what is known as “psychological depletion.” When a person must carefully account for every penny, the remaining mental energy is insufficient to sustain emotional stability and cognitive control. This depletion is not momentary but rather a continuous erosion of psychological capital. However, as individuals grapple with this scarcity-induced depletion, their health-related outcomes and subsequent coping strategies are often far more complex than a simple decline in well-being (3).

Beyond the psychological erosion explained by both theories, financial strain may infiltrate physical health through two primary pathways as suggested by Keese and Schmitz (4). First, chronic fiscal stressors can provoke psychosomatic conditions, where the internal tension of economic insecurity manifests as tangible physical deterioration and somatic pain. Second, under severe resource constraints, medical care and health protection are often deprioritized. This “budgetary trade-off” extends to nutritional choices, for instance, individuals facing financial strain may prefer energy-dense but nutrient-poor food, while avoiding pricier healthy alternatives (15). Beside the budgetary trade-off, early theories also suggested that smoking and other substance use function as mechanisms to minimize negative emotional reactions triggered by stress (16). Consequently, the impact of financial strain is not merely a fiscal barrier but a catalyst for a cluster of unhealthy behaviors (17, 18).

However, while extant theory primarily emphasizes the negative association of financial strain on health, the underlying causal mechanisms remain complex and contested. Many studies suggest that high levels of economic hardship push lower-income individuals into a cycle of illness, employment instability, and further financial decay, others raise the possibility of reverse causation (19). For instance, Iskander et al. (20) found evidence from rural poor Cambodians that poor health increases the probability of entering financial strain due to high medical expenditures which ultimately undermines health in the longer-term. The evidence regarding the behavioral link is similarly nuanced. While some research identifies significant correlations between financial strain and excessive drinking or smoking (21, 22), some analyses have failed to find robust associations (23). Some also argue financial strain and a healthy lifestyle seem to be partly mediated by self-control (24). Based on this background, we propose:

*Hypothesis 1 (H1): Financial strain is negatively associated with physical and psychological health status and is negatively related to proactive health-promoting behaviors*.

### 2.2 Financial strain and digital health triage

With the rapid development of digitalization, using digital tools to address health issues has become increasingly popular in many societies (9, 25). Researchers claim that the internet acts as a “Digital Dividend” providing a low-cost mechanism to bridge the gap in health resource accessibility (26). For those facing financial strain, the utilitarian motivation to use the internet for health purposes may intensify. The internet is not only a leisure tool, but a functional necessity used to mitigate the perceived threat of illness while bypassing the high costs of traditional medical consultations (9). Rather than serving as a mere supplement to professional care, digital health-seeking may function as a cost-mitigation strategy or a preparatory filtering mechanism (7). By utilizing digital resources to validate symptoms or seeking support, individuals facing financial strain may attempt to optimize their limited financial capital using information capital. Empirical studies have observed that individuals use online resources to validate symptoms and determine the severity of their condition before committing to the registration fees and transportation costs associated with a hospital visit (8). Thus, in this study we propose:

*Hypothesis 2 (H2): Financial strain is positively associated with the reliance on digital health information for medical decision-making*.

### 2.3 Financial strain and medical preferences: the role of TCM

In the Chinese healthcare system, TCM and Western Medicine represent two distinct but complementary systems (27). While Western Medicine is often associated with high-tech interventions and higher immediate costs, TCM is frequently perceived as a more holistic, preventative, and economically flexible option. As noted in research by Banerjee et al. (28) in rural India the poor frequently bypass formal, state-run health services in favor of informal providers or traditional healers. This preference is not necessarily a rejection of modern medicine but a rational response to the scarcity of accessible formal care. The informal healers and traditional practitioners are physically present in the community, socially integrated, and offer flexible payment structures. Thus, the association between financial strain and medical preferences may also be shaped by the interaction between cultural context and institutional factors.

In the Chinese context, many TCM services often serve a parallel function. The chronic stress of financial instability is associated with a greater appeal of medical systems that emphasize long-term maintenance rather than expensive, acute interventions. If financial strain limits access to high-cost specialist care, individuals may turn to TCM as a culturally familiar and perceived cost-effective alternative. Thus, health-seeking behavior is reshaped by a pragmatic search for accessible care that fits within narrowed economic and cognitive constraints. We therefore propose:

*Hypothesis 3 (H3): Financial strain is positively associated with both the cognitive preference for and the behavioral utilization of Traditional Chinese Medicine*.

## 3 Methodology

### 3.1 Data

The empirical analysis of this study utilizes data from the 2021 wave of the China General Social Survey (CGSS), conducted by the National Survey Research Center at Renmin University of China. The CGSS is the earliest nationwide, comprehensive, and continuous large-scale social survey project in China. It covers 28 provinces, autonomous regions, and municipalities across mainland China, ensuring a highly representative sample of the Chinese population.

The 2021 wave initially comprised a total sample of 8,148 respondents. Following the standard CGSS protocol, the 2021 questionnaire adopted a modular split-sample design. While the core questions were administered to the entire sample, specific thematic modules, such as the Health Module and the Specialized Social Identity Module, were administered to independent, randomly assigned sub-samples with over 2,000 respondents.

Consequently, the analytical sample for this study is derived from the intersection of the different modules. By merging samples from three different modules, we were able to generate two datasets. Dataset one includes the key variable financial strain, and some health-related variable, such as health-risk/promoting behaviors, somatic symptoms, and psychological distress. The data on TCM preferences, and digital health behaviors also included in Dataset one. Dataset two includes financial strain together with more granular indicators such as sleep quality, medical checkup frequency, physical/psychological impairment, and psychological well-being.

After merging these modules and excluding observations with missing values, “unknown,” or “refused” responses, the final analytical samples were determined. Dataset one includes 623 respondents, and Dataset two includes 592 respondents. A comparison between our sub-sample and the full CGSS 2021 sample reveals a higher concentration of the active labor force in our data (as detailed in Appendix Table A1). Accordingly, this sub-sample exhibits a higher employment rate and a younger age profile. While our sample may not fully represent the general population, this focus is theoretically justified, as primary household earners are most sensitive to economic fluctuations, making their lived experiences highly relevant to the study of financial strain and resource scarcity. To ensure the reliability of our estimates, we include a comprehensive set of demographic controls to account for compositional differences. Therefore, following Solon et al. (29), we prioritize unweighted OLS with robust standard errors to maintain statistical efficiency and avoid the variance inflation that would result from applying mismatched population weights to this specific sub-group.

### 3.2 Measurements

*Financial strain*. As a broad concept, financial strain is usually assessed through different measures that aim to evaluate specific aspects of financial pressure and resource scarcity (30). This study utilizes three items from the CGSS related to economic circumstances as present in Table 1. A composite index was created by averaging the standardized (z-scores) of these items (31). Higher scores indicate a more severe level of financial constraint.

**Table 1:** Measurements for financial strain and health related variables.

Variables	Measurements
Financial strain *(3 items)*	1. *Compared to last year, your family's income situation this year is…* 2. *Overall, what was the status of your household's income and expenditure over the past year?* 3. *Assuming that in the next month your household needs an expense of 10,000 Yuan, would you be able to pay it without borrowing?*
Self-rated health	*How would you rate your current overall physical health status? (+)*
Health related variables from Dataset One	
Health-risk behaviors *(2 items)*	1. *Do you smoke cigarettes? If yes, how many cigarettes per day on average? (+)* 2. *How often do you drink alcoholic? (+)*
Health-promoting behaviors *(2 items)*	1. *How often do you engage in physical exercise for at least 20 minutes that is intense enough to make you sweat or breathe harder? (+)* 2. *How often do you eat fresh fruits or vegetables? (+)*
Somatic symptoms *(2 items)*	1. *In the past four weeks, have you frequently experienced bodily pain? (+)* 2. *I often feel significant discomfort in certain parts of my body. (+)*
Psychological distress *(3 items)*	*In the past four weeks, have you frequently experienced any of the following problems?* 1. *Felt unhappy or depressed. (+)* 2. *Lost confidence in yourself. (+)* 3. *Felt unable to overcome your difficulties. (+)*
Health related variables from Dataset Two	
Subjective sleep quality *(1 item)*	1. *During the past month, how would you rate your sleep quality overall? (+)*
Emotional impairment *(2 items)*	*During the past 4 weeks, how much of the time have you had any of the following problems with your work or other regular daily activities as a result of any emotional problems (such as feeling depressed or anxious)?* 1. *Cut down on the amount of time you spend on work or other activities. (+)* 2. *Accomplished less than you would like. (+)*
Preventive health utilization *(1 item)*	1. *In the last three years, have you had any general health checkups (physical examination)? (+)*
Psychological well-being *(2 items)*	*These questions are about how you feel and how things have been with you during the past 4 weeks. For each question, please choose the one answer that comes closest to the way you have been feeling*. 1. *Have you felt calm and peaceful? (+)* 2. *Did you have a lot of energy? (+)*
Physical functioning*(3 items)*	1. *The following questions are about activities you might do during a typical day. Does your health now limit you in these activities? If so, how much? (1) Moderate activities, such as moving a table, pushing a vacuum cleaner, bowling, or playing golf. (+) (2) Climbing several flights of stairs. (+)* 2. *During the past 4 weeks, how much did pain interfere with your normal work (including both work outside the home and housework)? (–)*
Digital health behavior	
Lifestyles information mental health information	*During the past 12 months, how often have you used the internet to search for information on the following topics?* 1. *Information about healthy lifestyles. (+)* 2.*Information related to anxiety, stress, or similar conditions. (+)*
Decision support Advice validation Information reliability Information impact Communication bridge	*To what extent do you agree with the following statements?* 1. *The internet can help people decide whether they need to see a doctor. (+)* 2. *The internet helps confirm whether a doctor has given people appropriate advice. (+)* 3. *It is difficult to distinguish reliable health information from unreliable information online. (+)* 4. *In the past 12 months, information on the internet has had a positive impact on my health behaviors. (+)* 5. *In the past 12 months, information on the internet has helped me understand what my doctor told me. (+)*
TCM preference and utilization	
Medical preference	*To what extent do you agree with the following statement: Traditional Chinese Medicine is more effective than Western Medicine?*
Doctor visit (Western) Doctor visit (TCM)	*During the past 12 months, how often have you visited a doctor?* 1. *A doctor of Western Medicine. (+)* 2. *A doctor of Traditional Chinese Medicine. (+)*

“+” indicates that a higher score corresponds to a higher level of the construction (e.g., more frequent, better, or more agreement); “–” indicates the opposite.

*Health outcomes*. To capture the comprehensive relationships of financial strain, we categorize health outcomes into three primary domains, incorporating physical, psychological and behavioral dimensions:

i. Physical health and functional status: this includes *Self-rated Health* (a global measure of overall physical status), *Somatic Symptoms* (frequency of bodily pain and discomfort), *Physical Functioning* (limitations in daily activities and housework due to health issues).
ii. Psychological well-being and distress: in this dimension we assess *Psychological Distress* (negative effect and loss of confidence), *Psychological Well-being* (positive effect and vitality), and *Emotional Impairment* (the degree to which emotional problems reduce productivity or work time).
iii. Health behaviors and maintenance: this domain covers *Health-Risk Behaviors* (intensity of smoking and drinking), *Health-Promoting Behaviors* (physical exercise and produce intake), *Subjective Sleep Quality* and *Preventive Health Utilization* (frequency of general health checkups).

*Digital health behavior*. To explore the digital extension of health coping, we measure seven internet-based health behavior. These are categorized into:

i. Information seeking: the frequency of searching for information on healthy lifestyles and mental health (anxiety/stress).
ii. Perceived utility and trust: respondents' agreement with the internet's role in decision support, advice validation, and communication bridge.
iii. Appraisal and impact: the perceived difficulty in distinguishing reliable information and the positive impact of online information on health behaviors.

*TCM preference and utilization*. To capture the cultural-bound medical shift, we measure:

i. Cognitive preference: the extent to which respondents agree that TCM is more effective than Western Medicine.
ii. Utilization behavior: the actual frequency of visits to TCM doctors and Western medical doctors during the past 12 months.

All variables comprising multiple items were standardized and aggregated into composite indices by averaging their scores to ensure measurement reliability. Detailed question wording for all variables is presented in Table 1.

*Control variables*. To isolate the effect of financial strain, we control for a set of demographic and socioeconomic factors: gender, residence (urban/rural), age and age squared (to account for non-linear aging effects), education level, employment status, log-transformed household income, marital status, and the presence of any pre-existing long-term illness or disability.

### 3.3 Analytical strategy

To estimate the multi-dimensional association of financial strain with health outcomes and digital health behaviors, our primary empirical approach utilizes Ordinary Least Squares (OLS) regression models. The baseline model is specified as follows:


$$\begin{array}{l} Y_{i}=\beta_{0}+\beta_{1}FS_{i}+\gamma X_{i}+\epsilon_{i} \end{array}$$ (1)

Where *Y*_*i*_ represents the respective dependent variable (e.g., self-rated health, psychological distress, or digital health reliance) for individual *i*. *FS*_*i*_ denotes the primary independent variable, financial strain. *X*_*i*_ is a vector of demographic and socioeconomic control variables, and ϵ_*i*_ represents the error term. To address potential issues of heteroskedasticity, all models are estimated using robust standard errors. To ensure the validity of our estimates, we conducted multicollinearity diagnostics using the Variance Inflation Factor (VIF). The VIF values for all independent and control variables were strictly evaluated, confirming that multicollinearity does not pose a threat to our regression. results^1^

Furthermore, acknowledging the ordinal nature of several single-item dependent variables in our study, we conducted rigorous robustness checks using Ordered Logit (*ologit*) models. The results generated from the *ologit* specifications were highly consistent with our baseline OLS estimates in terms of both the direction of the coefficients and their statistical significance.

Finally, for the moderation analysis exploring possibility of digital resilience, we introduced interaction terms between financial strain and specific digital health behaviors. To prevent structural multicollinearity and facilitate the interpretation of the main effects within the interaction models, the continuous moderating variables were mean centered prior to generating the interaction terms. All data preparation and statistical analyses were conducted using Stata18.

## 4 Results

### 4.1 The multi-dimensional relationship between financial strain and health

To test Hypothesis 1, we examined the associations between financial strain and multiple health outcomes. Tables 2, 3 present the regression results from two independent modules of CGSS (Dataset One and Dataset Two), providing a validation of how financial strain associates with various dimensions of individual well-being in Chinese context. To providing baseline evidence, we conducted analysis from two datasets on the relationship between financial strain and self-rated overall health. As Column 1 in both Tables 2, 3 presents, financial strain exhibited a robust detrimental relationship. Higher levels of financial strain were significantly associated with lower Self-rated Health *(B* = −*0.206, p* < *0.01 in* *Table 2*and B = −*0.197, p* < *0.01 in* *Table 3*).

**Table 2:** Financial strain and health outcomes from dataset one.

Variables	(1)	(2)	(3)	(4)	(5)
	Self-rated health	Health-risk behaviors 1	Health-promoting behaviors	Somatic symptoms	Psychological distress
Financial strain	−0.206^***^ (0.0566)	0.0972 (0.0980)	−0.309^***^ (0.112)	0.302^***^ (0.0912)	0.189^***^ (0.0414)
Gender	0.0372 (0.0665)	1.845^***^ (0.117)	−0.0405 (0.143)	−0.204^*^ (0.110)	−0.130^**^ (0.0521)
Urban	−0.0195 (0.0766)	0.00212 (0.165)	−0.161 (0.172)	0.0741 (0.130)	−0.00215 (0.0583)
Age	−0.0538^***^ (0.0200)	0.116^***^ (0.0347)	0.0545 (0.0407)	0.0448 (0.0294)	0.0182 (0.0139)
Age2	0.000482^**^ (0.000229)	−0.00120^***^ (0.000391)	−0.000601 (0.000452)	−0.000564^*^ (0.000332)	−0.000361^**^ (0.000153)
Education	−0.0478 (0.0359)	−0.133^*^ (0.0763)	0.189^**^ (0.0812)	0.119^**^ (0.0590)	0.0297 (0.0283)
Employed	−0.0601 (0.171)	−0.111 (0.462)	0.254 (0.554)	−0.203 (0.334)	0.0852 (0.184)
Income (ln)	0.00653 (0.0168)	0.0448 (0.0280)	−0.0191 (0.0407)	0.00656 (0.0302)	0.0147 (0.0128)
Married	−0.000432 (0.0832)	−0.163 (0.149)	0.165 (0.178)	0.0655 (0.134)	−0.0880 (0.0633)
Illness	−0.715^***^ (0.0983)	0.00426 (0.210)	0.283 (0.207)	1.175^***^ (0.178)	0.276^***^ (0.0832)
Constant	5.414^***^ (0.454)	−0.153 (0.919)	5.832^***^ (1.098)	−1.859^**^ (0.732)	−0.424 (0.378)
Observations	623	618	623	623	623
R-squared	0.179	0.284	0.044	0.131	0.144

Robust standard errors in parentheses; ^***^p < 0.01, ^**^p < 0.05, ^*^p < 0.1.

**Table 3:** Financial strain and health outcomes from dataset two.

Variables	(1)	(2)	(3)	(4)	(5)	(6)
	Self-rated health	Subjective sleep quality	Preventive health utilization	Physical functioning	Emotional impairment	Psychological well-being
Financial strain	−0.197^***^ (0.0672)	−0.123^**^ (0.0479)	−0.0941^**^ (0.0444)	−0.0697^***^ (0.0204)	0.123^**^ (0.0492)	−0.201^***^ (0.0504)
Gender	−0.0270 (0.0888)	0.0872 (0.0569)	−0.172^***^ (0.0613)	0.0487^*^ (0.0286)	−0.0791 (0.0617)	0.00456 (0.0649)
Urban	0.290^***^ (0.107)	−0.00440 (0.0709)	−0.0858 (0.0754)	−0.0189 (0.0349)	0.0800 (0.0762)	0.0480 (0.0780)
Age	−0.0594^**^ (0.0255)	−0.0155 (0.0172)	−0.00347 (0.0191)	0.00704 (0.00968)	−0.0128 (0.0183)	−0.00177 (0.0187)
Age2	0.000676^**^ (0.000283)	0.000155 (0.000196)	8.25e−05 (0.000219)	−0.000139 (0.000108)	4.24e−05 (0.000205)	9.28e−05 (0.000206)
Education	0.0589 (0.0522)	−0.0417 (0.0337)	0.147^***^ (0.0345)	0.00817 (0.0151)	−0.0342 (0.0352)	−0.0106 (0.0361)
Employed	0.0290 (0.191)	0.303^**^ (0.143)	−0.0741 (0.141)	0.0435 (0.0744)	0.0726 (0.144)	0.131 (0.150)
Income (ln)	−0.00268 (0.0172)	−0.0177 (0.0161)	0.0404^***^ (0.0136)	−0.00497 (0.00577)	0.0116 (0.0171)	−0.0128 (0.0157)
Married	−0.142 (0.115)	0.0292 (0.0738)	0.0777 (0.0817)	0.107^**^ (0.0435)	−0.143^*^ (0.0844)	−0.0537 (0.0968)
Illness	−0.981^***^ (0.109)	−0.246^***^ (0.0754)	0.104 (0.0868)	−0.304^***^ (0.0526)	0.198^**^ (0.0904)	−0.313^***^ (0.0881)
Constant	4.237^***^ (0.595)	3.292^***^ (0.423)	1.486^***^ (0.415)	2.688^***^ (0.207)	2.152^***^ (0.411)	3.977^***^ (0.427)
Observations	592	592	592	592	592	592
R-squared	0.164	0.046	0.104	0.190	0.055	0.057

Robust standard errors in parentheses; ^***^p < 0.01, ^**^p < 0.05, ^*^p < 0.1.

Regarding other health-related outcomes, results from both datasets indicate that financial strain is significantly associated with a broad spectrum of health indicators among Chinese residents. In line with the majority of existing evidence, financial strain is negatively associated with psychological well-being (*B* = −*0.201, p*<*0.01*) and positively associated with psychological distress (*B* = *0.189, p*<*0.01*). Furthermore, our analysis extends current literature by examining emotional impairment, a dimension seldom addressed in previous studies. As shown in Table 3 (Column 5), individuals experiencing higher financial strain report a significantly greater frequency of emotional impairment (*B* = *0.123, p*<*0.05*).

For physical health, our evidence demonstrates that financial strain is significantly associated with an increased prevalence of somatic symptoms (*B* = *0.302, p*<*0.01*) and a decline in physical functioning (*B* = −*0.0697, p*<*0.01*). These findings suggest that economic scarcity manifests as tangible physical discomfort. Furthermore, financial strain is linked to poorer sleep quality (*B* = −*0.123, p*<*0.05*), indicating a persistent erosion of the psychological bandwidth necessary for physiological recovery.

In terms of health-related behaviors, financial strain significantly inhibited proactive health maintenance. Both participation in health-promoting behaviors (*B* = −*0.309, p*<*0.01*) and the frequency of preventive health utilization (*B* = −*0.0941, p*<*0.05*) declined as financial constraints intensified. Notably, contrary to some existing literature, health-risk behaviors (e.g., smoking and drinking) did not show a statistically significant relationship with financial strain in our sample (*B* = *0.0972, p* > *0.1*). This suggests that in the Chinese context, the health penalty associated with financial strain is more strongly linked to the depletion of health-promoting investments than to an escalation in maladaptive consumption. This decoupling may be attributed to the unique sociocultural role of tobacco and alcohol in China, where smoking and drinking function as deeply entrenched tools for social networking and the maintenance of “Guanxi” (32, 33).

In summary, the results in Tables 2, 3 provide consistent support for Hypothesis 1. Financial strain was significantly associated with poorer self-rated health, more somatic symptoms, lower psychological well-being, worse sleep quality, and reduced preventive health investments, while showing no significant association with health-risk behaviors.

Supportive findings also can be found from both Tables 2, 3 that income level is significantly associated only with preventive health utilization, but not with any of the other health-related outcomes. This indicates that, unlike the Stress Process Model suggests where financial strain is primarily viewed as a proxy for social stratification, financial strain in this context operates as a distinct and independent psychological stressor. Although income level reflects the material capacity for health-promoting investments, it does not fully capture the internal cognitive reality of economic hardship (34). Our results suggest that the pervasive health penalties are more strongly associated with the subjective experience of scarcity. In other words, the physiological toll of financial pressure remains a more consistent predictor of health erosion than the mere lack of financial resources.

Moreover, across both tables, age and age-square typically displayed a non-linear relationship with health. Specifically, for Self-Rated Health, the negative coefficient for age and the positive coefficient for age-squared suggest a U-shaped relationship. Gender differences were also significant. In Dataset One (Table 2), males reported higher health-risk behaviors, but significantly lower levels of somatic symptoms and psychological distress compared to females. Education served as a robust protective factor, correlating positively with health-promoting behaviors (Table 2) and preventive health utilization (Table 3), while negatively correlating with somatic symptoms. As expected, the presence of Chronic Illness (*Illness*) was one of the most powerful predictors across all models. It was consistently associated with lower Self-rated Health, poorer sleep quality, and higher levels of somatic symptoms, emotional impairment, and psychological distress.

### 4.2 Financial strain and digital health strategies: seeking and reliance

Table 4 examines the associations between financial strain and internet-based health behaviors, as proposed in Hypothesis 2. Showing in columns (1) and (2), financial strain is significantly associated with a higher frequency of searching for mental health information (*B* = *0.119, p*<*0.05*), while its effect on general lifestyle information remains negligible. Regarding decision support and information appraisal, a distinct pattern of “digital triage” emerges. Respondents facing higher financial strain are significantly more likely to rely on the internet to decide whether to seek professional medical consultation (*B* = −*0.116, p*<*0.05*). Intriguingly, they are also significantly less likely to perceive difficulty in distinguishing reliable health information online (*B* = *0.130, p*<*0.05*). These results suggest that financial scarcity is related to an adaptive, yet subjectively confident, reliance on digital resources. One interpretation is that the urgent need for low-cost solutions may lead individuals to perceive less difficulty in navigating complex online health information. The lack of significant findings regarding advice validation and communication bridge suggests that while the internet serves as a critical gatekeeper for the resource-constrained it has not yet fully replaced the formal channels of medical interaction for deciding how to engage with providers.

**Table 4:** Financial strain and internet usage on health issues.

Variables	(1)	(2)	(3)	(4)	(5)	(6)	(7)
	Lifestyles information	Mental health information	Decision support	Advice validation	Communication bridge	Information reliability	Information impact
Financial strain	−0.0786 (0.0580)	0.119^**^ (0.0511)	0.116^**^ (0.0586)	0.0208 (0.0524)	0.00477 (0.0553)	−0.130^**^ (0.0533)	−0.00916 (0.0575)
Controls	Yes	Yes	Yes	Yes	Yes	Yes	Yes
Observations	623	623	602	601	568	601	580
R-squared	0.178	0.119	0.015	0.020	0.031	0.066	0.052

Robust standard errors in parentheses; ^***^p < 0.01, ^**^p < 0.05, ^*^p < 0.1.

Moreover, the prevalence of these digital strategies raises a critical question regarding their potential moderating role in health. To explore whether certain digital engagement patterns function as a buffering effect, we examined the moderating effects of all available internet behaviors on the relationship between financial strain and health outcomes. The results reveal that only specific forms of digital engagement function as buffering mechanisms (Table 5). First, perceived information efficacy significantly mitigates psychological erosion, for individuals who believe online information positively relates to their health behaviors. The association between financial strain and psychological distress is significantly weakened (*B* = *0.0866, p*<*0.05*). Second, among respondents under high financial strain, using the internet to confirm a doctor's advice significantly mitigates the decline in self-rated health (*B* = −*0.127, p*<*0.05*). This suggests that internet-based medical validation may play a protective role for subjective overall health. Finally, active searching for mental health information functions as a marginal buffer (*B* = −*0.0724, p* = *0.079*), suggesting a potential role that warrants further investigation.

**Table 5:** Moderating effect of internet usage.

Variables	(1)	(2)	(3)
	Psychological distress	Self-rated health	Psychological distress
Financial strain	0.291^***^ (0.0871)	−0.208^***^ (0.0579)	0.158^***^ (0.0386)
Mental health information	0.198^***^ (0.0282)		
Mental health information ^*^ Financial strain	−0.0724^*^ (0.0411)		
Advice validation		0.0533 (0.0388)	
Advice validation ^*^ Financial strain		−0.127^**^ (0.0590)	
Information impact			0.0428 (0.0298)
Information impact ^*^ Financial strain			0.0866^**^ (0.0415)
Controls	Yes	Yes	Yes
Observations	620	601	575
R-squared	0.213	0.182	0.136

Robust standard errors in parentheses ^***^p < 0.01, ^**^p < 0.05, ^*^p < 0.1.

Overall, the above findings largely support our Hypothesis 2, showing that financial strain is associated with a greater reliance on the internet for deciding whether to seek professional care, a “digital triage” strategy. However, not all digital behaviors were equally affected; the null findings for lifestyle information searching and advice validation suggest that digital reliance is domain specific.

### 4.3 TCM as an alternative

In line with Hypothesis 3, Table 6 examines healthcare preferences among Chinese residents facing financial scarcity. In terms of utilization behavior, financial strain displayed divergent associations with Western vs. TCM. While financial strain had no significant relationship with the frequency of Western Medical Visits (*B* = *0.00423, p* > *0.1*), it is significantly associated with the frequency of visits to TCM doctors (*B* = *0.101, p*<*0.05*). This utilization pattern also aligns with cognitive preferences: financial strain was significantly associated with a stronger belief that “Traditional Chinese Medicine is more effective than Western Medicine” (*B* = *0.124, p*<*0.05*). These results indicate that rather than a simple withdrawal from all medical services, financial strain is associated with a preference for TCM over Western medicine. TCM, with its cultural resonance and perceived economic flexibility, may serve as a more accessible option for those who perceive formal Western medical care as posing a prohibitive financial or cognitive burden. Consistent with the “tunneling effect” of scarcity, individuals may not simply abandon health maintenance but instead are more likely to turn to systems of care that align more closely with their available economic and cultural capital.

**Table 6:** Financial strain and alternative medical choices.

Variables	(1)	(2)	(3)
	TCM preference	Western visit	TCM visit
Financial strain	0.124^**^ (0.0524)	0.00423 (0.0527)	0.101^**^ (0.0511)
Controls	Yes	Yes	Yes
Observations	622	623	623
R-squared	0.034	0.077	0.077

Robust standard errors in parentheses; ^***^p < 0.01, ^**^p < 0.05, ^*^p < 0.1.

## 5 Discussion

This study provides a holistic examination of the multi-dimensional health penalties of financial strain while revealing unique coping patterns in the digital and cultural spheres. Our findings suggest that financial strain may act as a stressor that erodes physical functioning, psychological well-being, and preventive investments. Crucially, we identify that individuals under financial constraint do not simply withdraw from healthcare; instead, they are more likely to rely on internet-based triage and Traditional Chinese Medicine (TCM) as accessible alternatives to the formal Western medical system for residents in China.

The negative association between financial strain with self-rated health, somatic symptoms, and sleep quality aligns with the Scarcity Theory (14) and the Stress Process Model (13). Furthermore, our results regarding the reduction in formal preventive health utilization corroborate empirical evidence from Western contexts, such as Kalousova and Burgard (35), who demonstrated that highly indebted households frequently forego necessary medical and dental care as a primary strategy to manage resource scarcity. Interestingly, the lack of association between financial strain and health-risk behaviors (smoking and drinking) provides a nuanced counterpoint to Western-centric literature (18), which often links poverty to increased substance use. In the Chinese context, the health decline observed here appears to be more strongly associated with the erosion of health-promoting investments than with the escalation of risky consumption.

A key contribution of this study is the revelation of digital behaviors. Traditional ‘digital divide' frameworks often posit that socioeconomically disadvantaged groups lack the digital literacy or access to utilize online health resources effectively [e.g., (36)]. In contrast, our findings suggest that individuals facing financial strain were significantly more likely to use the internet to decide whether a professional medical visit is necessary. Within the framework of resource scarcity, the internet is not merely an information source but a cost-saving gatekeeper. The most striking finding is that financially strained individuals perceive less difficulty in distinguishing online information reliability. This suggests a potential cognitive pattern. When formal healthcare is financially out of reach, the scarcity mindset may lead individuals to perceive online advice as highly useful while reporting less concern about its risks. This creates a potential risk where the most economically at-risk populations are also the most likely to rely on potentially unverified digital health information while perceiving little difficulty in doing so.

Finally, previous literature has revealed that in some developing countries, financially constrained populations often turn to traditional or informal healthcare providers to optimize limited resources (28). Our empirical results confirm that in the Chinese context, financial strain is significantly associated with both the belief in TCM efficacy and the frequency of TCM visits, while exhibiting no inhibitory effect on Western medical visits. This indicates that TCM serves as a complementary alternative specifically tailored to resource-constrained realities. This pattern likely reflects the fact that TCM is widely perceived in China as more economically flexible and accessible compared to Western medicine (11). Furthermore, the cultural resonance of TCM may offer a sense of familiarity and control that could be particularly appealing to individuals feeling alienated by the perceived high costs and profit-driven nature of formal hospital systems.

## 6 Limitations

Despite its contributions, this study has several limitations that warrant acknowledgment. First, the use of cross-sectional data from the CGSS 2021 precludes the establishment of definitive causal relationships. While Scarcity Theory suggests that financial strain precedes health erosion and shifts in medical strategy, longitudinal data would be required to track these transitions over time and account for potential bi-directional effects.

Second, the sample size was restricted by the modular split-sample design of the CGSS. Although we clarify that our sample limits generalizability to the older population or unemployed, it provides a useful lens into the financial strain and digital coping mechanisms of working-age population. Future research with larger integrated datasets could provide more granular insights into how these patterns vary across different age cohorts or geographic regions in China.

Third, while we identified a significant association with TCM visit, our data does not allow for a detailed cost-benefit analysis of TCM vs. Western medical treatments for specific conditions. Future qualitative research could explore the lived experiences of financially strained individuals to better understand whether the preference for TCM is driven primarily by perceived cost, cultural resonance, or a combination of both.

Finally, while our study highlights a “digital triage” strategy, we did not measure the clinical accuracy of the information retrieved. The potential for misdiagnosis through over-reliance on digital resources remains a critical area for future investigation, particularly regarding its long-term impact on health outcomes for socioeconomically vulnerable groups.

## 7 Conclusion

This study provides a multi-dimensional examination of the relationship between financial strain and health and health-seeking behaviors within the Chinese context. By moving beyond a simple stress-to-health correlation, we have found that financial strain may act as a systemic force that not only compromises physical and psychological well-being but also influences the individual's medical decision-making landscape. The three hypotheses proposed in this study were all supported. Financial strain was associated with multidimensional health penalties (H1), a distinct pattern of digital health triage (H2), and an association in medical preferences with TCM (H3).

Our findings yield three primary conclusions. First, we provide evidence that financial strain is a pervasive stressor that infiltrates multiple domains of health and is associated with significantly lower preventative health investments. Second, in the digital sphere, financial strain is closely linked to a unique reliance on internet-based health resources. Individuals facing financial constraints tend to rely on digital triage, using the internet to make medical decisions, often accompanied by a lack of regard for information reliability. Third, our analysis reveals a significant pattern in healthcare utilization. Financial strain is significantly associated with a stronger cognitive preference for and greater behavioral utilization of TCM. This suggests that TCM serves as a culturally resonant and economically adaptive alternative when formal Western medical services are perceived as financially burdensome.

These results have critical implications for health policy and equity. True health equity requires more than just clinical interventions or basic medical insurance. It necessitates addressing the underlying scarcity mindset that may drive vulnerable populations toward fragmented digital information and alternative medical paths. Future interventions should focus on enhancing digital health literacy and integrating culturally trusted systems like TCM into formal primary care to ensure that those under financial strain are not marginalized into lower-quality or unverified health-seeking pathways. By understanding these adaptive strategies, policymakers can better design mechanisms that preserve the cognitive bandwidth and health capital of the most economically vulnerable citizens.

## Data Availability

Publicly available datasets were analyzed in this study. This data can be found here: https://www.cnsda.org/index.php?r=site/datarecommendation.

## Footnotes

1 The VIF for our primary independent variable, financial strain, is 1.16, and the VIFs for all other demographic and socioeconomic controls (gender, urban, income, education, etc.) are all below 1.5, far below the conservative threshold of 10. While the VIFs for age and age^2^ are elevated (approx. 54.4 and 52.5), this is a classic case of structural multicollinearity inherent in polynomial regression models. As noted in econometric literature [e.g., (37)], such correlation between a variable and its quadratic term is harmless; it does not introduce bias into the coefficients or affect the significance of other independent variables in the model.

## References

[1] Bosworth B. Increasing disparities in mortality by socioeconomic status. Annu Rev Public Health. (2018) 39:237–51. doi: 10.1146/annurev-publhealth-040617-014615 29608870

[2] Pathirana TI Jackson CA. Socioeconomic status and multimorbidity: a systematic review and meta-analysis. Aust N Z J Public Health. (2018) 42:186–94. doi: 10.1111/1753-6405.12762 29442409

[3] French D Vigne S. The causes and consequences of household financial strain: a systematic review. Int Rev Financ Anal. (2018) 62:150–6. doi: 10.1016/j.irfa.2018.09.008

[4] Keese M Schmitz H. Broke, Ill, and obese: is there an effect of household debt on health? Rev Income Wealth. (2014) 60:525–41. doi: 10.1111/roiw.12002

[5] Richardson T Elliott P Roberts R. The relationship between personal unsecured debt and mental and physical health: a systematic review and meta-analysis. Clin Psychol Rev. (2013) 33:1148–62. doi: 10.1016/j.cpr.2013.08.009 24121465

[6] Turunen E Hiilamo H. Health effects of indebtedness: a systematic review. BMC Public Health. (2014) 14:489. doi: 10.1186/1471-2458-14-489 24885280 PMC4060868

[7] Renahy E Chauvin P. Internet uses for health information seeking. Rev Épidémiol Santé Publique. (2006) 54:263–75. doi: 10.1016/S0398-7620(06)76721-9 16902386

[8] Alghamdi KM Moussa NA. Internet use by the public to search for health-related information. Int J Med Inform. (2012) 81:363–73. doi: 10.1016/j.ijmedinf.2011.12.004 22217800

[9] Bujnowska-Fedak MM. Trends in the use of the internet for health purposes in Poland. BMC Public Health. (2015) 15:194. doi: 10.1186/s12889-015-1473-3 25886280 PMC4349300

[10] Zhang D Zhang G Jiao Y Wang Y Wang P. “Digital dividend” or “Digital divide”: what role does the internet play in the health inequalities among Chinese residents? Int J Environ Res Public Health. (2022) 19:15162. doi: 10.3390/ijerph192215162 36429878 PMC9690004

[11] He P Zhu D Man X Bai Q Huang L Shi X . Strengthening of traditional Chinese medicine in the health system reform: effect on health outcomes and financial protection. Evid Based Complement Alternat Med. (2022) 2022:7226674. doi: 10.1155/2022/7226674 35096115 PMC8791714

[12] Kahn JR Pearlin LI. Financial strain over the life course and health among older adults. J Health Soc Behav. (2006) 47:17–31. doi: 10.1177/002214650604700102 16583773

[13] Pearlin L. The sociological study of stress. J Health Soc Behav. (1989) 30:241–56. doi: 10.2307/2136956 2674272

[14] Mani A Mullainathan S Shafir E Zhao J. Poverty impedes cognitive function. Science. (2013) 341:976–80. doi: 10.1126/science.1238041 23990553

[15] Neill BO Xiao JJ Sorhaindo B Garman ET. Financially distressed consumers: their financial practices, financial well-being, and health. J Financ Couns Plan. (2005) 16:73–87. Retrieved from: https://digitalcommons.uri.edu/hdf_facpubs/8/

[16] Tomkins SS. Understanding of smoking behavior. Am J Public Health Nations Health. (1966) 56:17–20. doi: 10.2105/AJPH.56.12_Suppl.17 PMC1267241 5951586

[17] Berg CJ Sanem JR Lust AK Ahluwalia JS Kirch MA An LC. Health-related characteristics and incurring credit card debt as problem behaviors among college students. Int J Mental Health. (2010) 6. doi: 10.5580/96d

[18] Grafova IB. Financial strain and smoking. J Fam Econ Issues. (2011) 32:327–40. doi: 10.1007/s10834-011-9247-2

[19] Lyons AC Yilmazer T. Health and financial strain: evidence from the survey of consumer finances. Southern Econ J. (2005) 71:873–90. doi: 10.2307/20062085

[20] Iskander D Picchioni F Zanello G Guermond V Brickell K. Sick of debt: how over-indebtedness is hampering health in rural Cambodia. Soc Sci Med. (2025) 367:117678. doi: 10.1016/j.socscimed.2025.117678 39884086

[21] Kendzor DE Businelle MS Costello TJ Castro Y Reitzel LR Cofta-Woerpel LM . Financial strain and smoking cessation among racially/ethnically diverse smokers. Am J Public Health. (2010) 100:702–6. doi: 10.2105/AJPH.2009.172676 20167886 PMC2836332

[22] Widome R Joseph AM Hammett P Van Ryn M Nelson DB Nyman JA . Associations between smoking behaviors and financial stress among low-income smokers. Prev Med Rep. (2015) 2:911–5. doi: 10.1016/j.pmedr.2015.10.011 26844167 PMC4721304

[23] Prentice C McKillop D French D. How financial strain affects health: evidence from the Dutch national bank household survey. Soc Sci Med. (2017) 178:127–35. doi: 10.1016/j.socscimed.2017.02.006 28214723

[24] Beenackers MA Groeniger JO Van Lenthe FJ Kamphuis CBM. The role of financial strain and self-control in explaining health behaviours: the GLOBE study. Eur J Public Health. (2018) 28:597–603. doi: 10.1093/eurpub/ckx212 29236973 PMC6051441

[25] Sumaedi S Sumardjo. Factors influencing internet usage for health purposes. Int J Health Gov. (2020) 25:205–221. doi: 10.1108/IJHG-01-2020-0002

[26] Zhong M Qiang D Wang J Sun W. Improving health and reducing health inequality: an innovation of digitalization? Soc Sci Med. (2024) 348:116847. doi: 10.1016/j.socscimed.2024.116847 38569285

[27] Keji C Hao X. The integration of traditional Chinese medicine and Western medicine. Eur Rev. (2003) 11:225–35. doi: 10.1017/S106279870300022X

[28] Banerjee A Deaton A Duflo E. Wealth, health, and health services in rural Rajasthan. Am Econ Rev. (2004) 94:326–30. doi: 10.1257/0002828041301902 19305517 PMC2658609

[29] Solon G Haider SJ Wooldridge JM. What are we weighting for? J Hum Resour. (2015) 50:301–16. doi: 10.3368/jhr.50.2.301

[30] Glei DA Goldman N Weinstein M. Perception has its own reality: subjective versus objective measures of economic distress. Popul Dev Rev. (2019) 44:1–27. doi: 10.1111/padr.12183 PMC6395043 30828111

[31] Logio K Dowdall G Babbie E Halley F. Creating composite measures. In: Adventures in Criminal Justice Research. Thousand Oaks, CA: Sage Publishing (2014), p. 74–95.

[32] Ma GX Shive SE Ma XS Toubbeh JI Tan Y Lan YJ . Social influences on cigarette smoking among Mainland Chinese and Chinese Americans: a comparative study. Am J Health Stud. (2013) 28:12–20. 24511220 PMC3914219

[33] Shell DF Newman IM Xiaoyi F. The influence of cultural orientation, alcohol expectancies and self-efficacy on adolescent drinking behavior in Beijing. Addiction. (2010) 105:1608–15. doi: 10.1111/j.1360-0443.2010.03006.x 20626375

[34] Zimmerman FJ Katon W. Socioeconomic status, depression disparities, and financial strain: what lies behind the income-depression relationship? Health Econ. (2005) 14:1197–215. doi: 10.1002/hec.1011 15945040

[35] Kalousova L Burgard SA. Debt and foregone medical care. J Health Soc Behav. (2013) 54:204–20. doi: 10.1177/0022146513483772 23620501

[36] Viswanath K Nagler RH Bigman-Galimore CA McCauley MP Jung M Ramanadhan S. The communications revolution and health inequalities in the 21st century: implications for cancer control. Cancer Epidemiol Biomarkers Prev. (2012) 21:1701–8. doi: 10.1158/1055-9965.EPI-12-0852 23045545 PMC3468900

[37] Allison PD. Multiple Regression: A Primer. Pine Forge Press (1999).

