# Supplementary material for: Financial strain, digital health reliance, and medical preferences: a multi-dimensional analysis of Chinese residents
Source: Front Public Health. 2026 May 20;14:1829939. doi: 10.3389/fpubh.2026.1829939 (PMC13230075; doi:10.3389/fpubh.2026.1829939)
Supplement: Supplementary file 1 [file Table_1.docx]

**Financial Strain, Digital Health Reliance, and Medical Preferences: A Multi-dimensional Analysis of Chinese Residents**

**Appendix**

Table A1 Demographic characteristics for the full sample of 2021 CGSS

| Variables | Obs | | Mean | | Std. dev. | Min | | Max |
| --- | --- | --- | --- | --- | --- | --- | --- | --- |
| Financial strain | 1,929 | | 5.5853 | | 1.5172 | 3 | | 9 |
| Self-rated health | 8,142 | | 3.4816 | | 1.0932 | 1 | | 5 |
| Income (ln) | 7,323 | | 8.0714 | | 4.2207 | 0 | | 13.816 |
| Age | 8,148 | | 51.6437 | | 17.5739 | 18 | | 99 |
| Variables | |  | | Freq. | | | Percent | |
| Gender | | Male | | 3,679 | | | 45.15 | |
|  | | Female | | 4,469 | | | 54.85 | |
| Urban | | Rural | | 4,574 | | | 56.14 | |
|  | | Urban | | 3,574 | | | 43.86 | |
| Education | | Primary and below | | 2,656 | | | 32.68 | |
|  | | Secondary | | 2,311 | | | 28.44 | |
|  | | High School | | 1,489 | | | 18.32 | |
|  | | College and above | | 1,671 | | | 20.56 | |
| Employed | | Employed | | 3,886 | | | 47.69 | |
|  | | Not Employed | | 4,262 | | | 52.31 | |
| Married | | Married | | 5,794 | | | 71.11 | |
|  | | Others | | 2,354 | | | 28.89 | |

Table A2 Demographic characteristics for the full sample of Dataset one

| Variables | Obs | | Mean | | Std. dev. | Min | | Max |
| --- | --- | --- | --- | --- | --- | --- | --- | --- |
| Financial strain | 623 | | 5.5634 | | 1.4880 | 3 | | 9 |
| Self-rated health | 623 | | 3.8475 | | 0.8771 | 1 | | 5 |
| Income (ln) | 623 | | 10.4385 | | 2.1793 | 0 | | 13.816 |
| Age | 623 | | 41.5056 | | 12.4040 | 18 | | 77 |
| Variables | |  | | Freq. | | | Percent | |
| Gender | | Male | | 351 | | | 56.34 | |
|  | | Female | | 272 | | | 43.66 | |
| Urban/Rural | | Rural | | 460 | | | 73.84 | |
|  | | Urban | | 163 | | | 26.16 | |
| Education | | Primary and below | | 70 | | | 11.24 | |
|  | | Secondary | | 169 | | | 27.13 | |
|  | | High School | | 138 | | | 22.15 | |
|  | | College and above | | 246 | | | 39.49 | |
| Employment | | Employed | | 607 | | | 97.43 | |
|  | | Not Employed | | 16 | | | 2.57 | |
| Marriage status | | Married | | 449 | | | 72.07 | |
|  | | Others | | 174 | | | 27.93 | |

Table A3 Demographic characteristics for the full sample of Dataset two

| Variables | Obs | | Mean | | Std. dev. | Min | | Max |
| --- | --- | --- | --- | --- | --- | --- | --- | --- |
| Financial strain | 592 | | 5.5473 | | 1.5417 | 3 | | 9 |
| Self-rated health | 592 | | 3.8260 | | 0.8461 | 1 | | 5 |
| Income (ln) | 592 | | 10.4132 | | 2.1432 | 0 | | 13.710 |
| Age | 592 | | 41.6453 | | 11.9346 | 19 | | 73 |
| Variables | |  | | Freq. | | | Percent | |
| Gender | | Male | | 291 | | | 49.16 | |
|  | | Female | | 301 | | | 50.84 | |
| Urban/Rural | | Urban | | 452 | | | 76.35 | |
|  | | Rural | | 140 | | | 23.65 | |
| Education | | Primary and below | | 72 | | | 12.16 | |
|  | | Secondary | | 149 | | | 25.17 | |
|  | | High School | | 121 | | | 20.44 | |
|  | | College and above | | 250 | | | 42.23 | |
| Employment | | Employed | | 566 | | | 95.61 | |
|  | | Not Employed | | 26 | | | 4.39 | |
| Marriage status | | Married | | 451 | | | 76.18 | |
|  | | Others | | 141 | | | 23.82 | |
